# Supplementary material for: Functional Fiber Reduces Mice Obesity by Regulating Intestinal Microbiota
Source: Nutrients. 2022 Jun 28;14(13):2676. doi: 10.3390/nu14132676 (PMC9268532; doi:10.3390/nu14132676)
Supplement: Supplementary file 1 [file nutrients-14-02676-s001.zip › nutrients-1740402-supplementary.pdf]

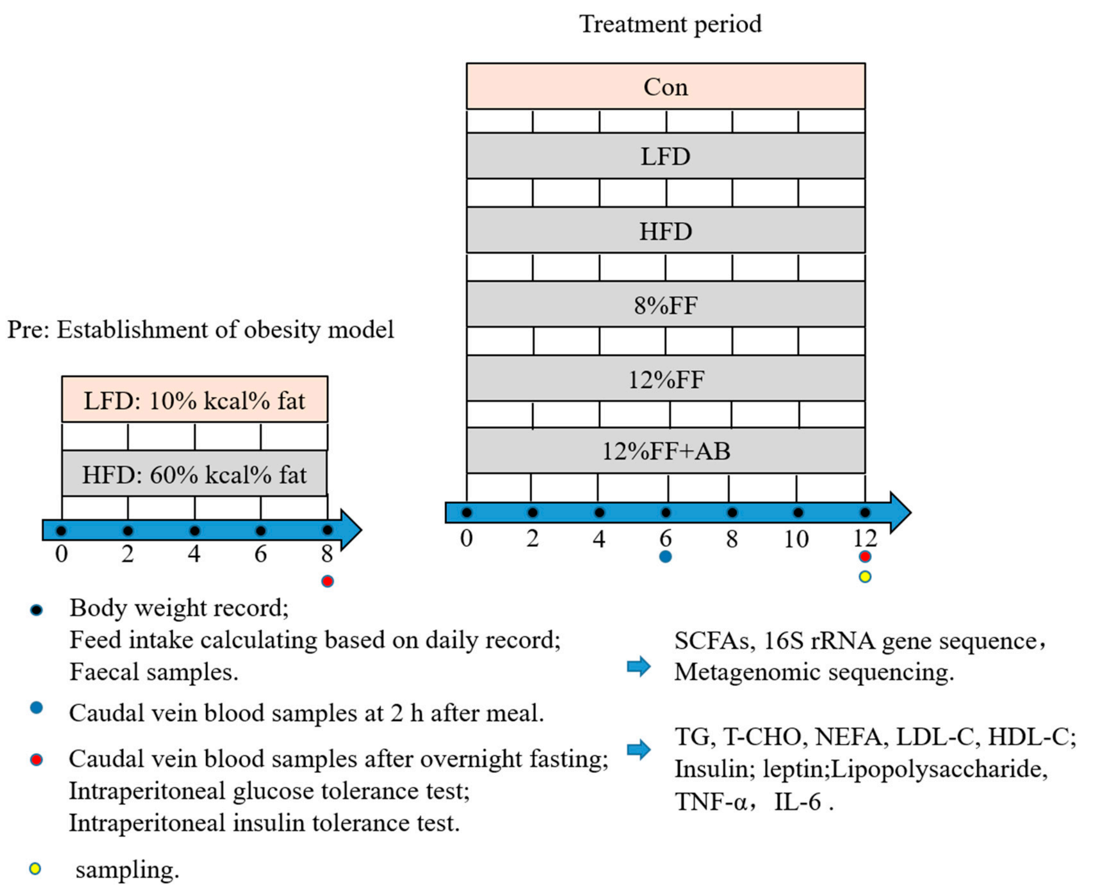

Figure S1. Overview of the study design and sample collection.

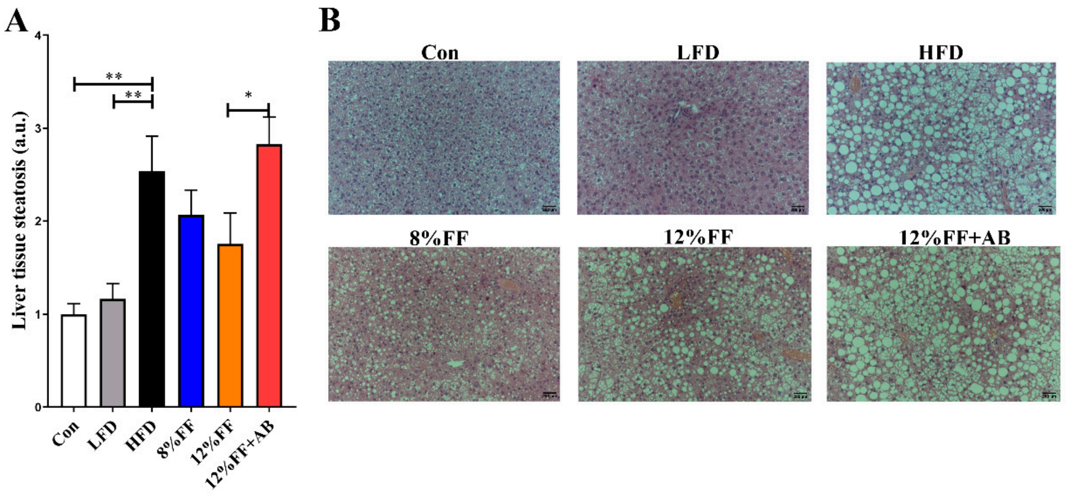

Figure S2 Effects of functional fiber on liver in obese mice. (A, B) H&E staining analysis of the effects of functional fiber on HFD-induced liver steatosis(n=7). The results are shown as the mean ± SEM. Significant differences are expressed as * ($p < 0.05$), and extremely significant differences are expressed as ** ($p < 0.01$).

Table S1. Experimental dietary formula

Group	LFD	HFD	HF+8%FF	HF+12%FF
Basal diet	TP23302	TP23300		
	low fat diet	high fat diet		
Kcal/gm	3.60	5.00		
Protein, kcal%	19	19.4		
Carbohydrate, kcal%	71	20.6		
Fat, kcal%	10	60		
Casein, gm	191.00	267.00	267.00	267.00
Corn starch, gm	497.00	0.00	0.00	0.00
Maltodextrin, gm	112.00	157.00	144.00	104.00
Sucrose, gm	64.00	89.00	89.00	89.00
Fat (Soybean oil + Lard), gm	40.00	334.00	334.00	334.00
Cellulose, gm	48.00	67.00	0.00	0.00
CSF, gm, gm	0.00	0.00	80.00	120.00
Vitamin Mix, V1010 and Mineral Mix, M1020, gm	43.00	79.00	79.00	79.00
L-Cystine, gm	3.00	4.00	4.00	4.00
Choline Bitartrate, gm	2.00	3.00	3.00	3.00
TBHQ, gm	0.008	0.067	0.067	0.067
Total, gm	1000.008	1000.067	1000.067	1000.067
Laboratory analysis				
Gross energy, kcal/gm	4.17	5.77	5.79	5.81

Table S2. Key Resource Table-supplemental oligonucleotides.

Gene Name	Primers 5’-3’
Mmu-β-actin	F: CACGATGGAGGGGCCGGACTCATC
	R: TAAAGACCTCTATGCCAACACAGT
Mmu-SCD1	F: TTCTTGCGATACACTCTGGTGC
	R: CGGGATTGAATGTTCTTGTCGT
Mmu-SREBP-1C	F: CTTTGGCCTCGCTTTTCGG
	R: TGGGTCCAATTAGAGCCATCTC
Mmu-ACC1	F: ATGGGCGGAATGGTCTCTTTC
	R: TGGGGACCTTGTCTTCATCAT
Mmu- PPARα	F: AGAGCCCCATCTGTCCTCTC
	R: ACTGGTAGTCTGCAAAACCAAA
Mmu-Fas	F: TATCAAGGAGGCCCATTTTGC
	R: TGTTTCCACCTCTAAACCATGCT
Mmu-CD36	F: GGAACTGTGGGCTCATTGC
	R: CATGAGAATGCCTCCAAACAC
Mmu-CPT1β	F: GGCACCTCTTCTGCCTTTAC
	R: TTTGGGTCAAACATGCAGAT
Mmu-UCP1	F: AGGCTTCCAGTACCATTAGGT
	R: CTGAGTGAGGCAAAGCTGATTT
Mmu-DIO2	F: CAAACAGGTTAAACTGGGTGAAGAT
	R: TCAGGTGGCTGAACCAAAGT
Mmu-Claudin-1	F: ATTTACTCCTATGCCGGCGA
	R: ACCTCATCGTCTTCCAAGCA
Mmu-Occludin	F: TGCATGTTCGACCAATGC
	R: AAGCCACTTCCTCCATAAGG
